# The Sensory Histidine Kinases TorS and EvgS Tend to Form Clusters in *Escherichia coli* Cells 

**DOI:** 10.1371/journal.pone.0077708

**Published:** 2013-10-11

**Authors:** Erik Sommer, Moriah Koler, Vered Frank, Victor Sourjik, Ady Vaknin

**Affiliations:** 1 Department of Molecular Biology, University of Heidelberg, Heidelberg, Germany; 2 Racah Institute of Physics, the Hebrew University, Jerusalem, Israel; University of Illinois at Urbana-Champaign, United States of America

## Abstract

Microorganisms use multiple two-component sensory systems to detect changes in their environment and elicit physiological responses. Despite their wide spread and importance, the intracellular organization of two-component sensory proteins in bacteria remains little investigated. A notable exception is the well-studied clustering of the chemoreceptor-kinase complexes that mediate chemotaxis behaviour. However, these chemosensory complexes differ fundamentally from other systems, both structurally and functionally. Therefore, studying the organization of typical sensory kinases in bacteria is essential for understanding the general role of receptor clustering in bacterial sensory signalling. Here, by studying mYFP-tagged sensory kinases in *Escherichia coli*, we show that the tagged TorS and EvgS sensors have a clear tendency for self-association and clustering. These sensors clustered even when expressed at a level of a few hundred copies per cell. Moreover, the mYFP-tagged response regulator TorR showed clear TorS-dependent clustering, indicating that untagged TorS sensors also tend to form clusters. We also provide evidence for the functionality of these tagged sensors. Experiments with truncated TorS or EvgS proteins suggested that clustering of EvgS sensors depends on the cytoplasmic part of the protein, whereas clustering of TorS sensors can be potentially mediated by the periplasmic/transmembrane domain. Overall, these findings support the notion that sensor clustering plays a role in bacterial sensory signalling beyond chemotaxis.

## Introduction

‘Two-component’ (TC) sensory systems are widespread in bacteria. Bacterial cells use these systems to sense environmental stimuli and elicit an appropriate adaptive cellular response [1–5]. A canonical TC system consists of two proteins: a sensory histidine kinase (HK) and a response regulator (RR). The sensory kinases are mostly membrane-bound receptors that sense different cues in the environment and communicate this information to their cytoplasmic kinase domain to control its rate of auto-phosphorylation at a conserved histidyl residue. The phosphoryl group can be then transferred to an aspartyl residue of the cognate response regulator. The response regulator protein is typically a transcription factor consisting of two domains: a receiver or regulatory domain that is phosphorylated by the kinase and an output domain that binds DNA [6,7]. The phosphorylation state of the receiver domain modifies the affinity of the binding domain for the DNA and thereby regulates expression of specific genes to elicit cellular responses. Various modifications of this scheme are found. For example, some sensors have dual function as kinase and phosphatase [8], which has been considered as a mechanism to generate a robust output [9,10]. Other systems, including the TorS and EvgS sensors, have multiple phosphotransfer steps within the sensor or between additional cytoplasmic proteins [11].

A unique member of the two-component family is the chemosensory system [12,13], which presumably evolved from the basic two-component scheme [2,14]. The chemosensory system controls the swimming behaviour of the bacterium with great sensitivity, large dynamic range, and fast response time [15]. In the chemosensory system, the sensing and the kinase functions are partitioned between two separated proteins: a transmembrane receptor and an associated cytoplasmic histidine kinase. The chemoreceptor/kinase complexes, together with a linker protein CheW, form tight hexagonal arrays, or clusters, located at the cell poles or along the cell body [16–19]. Allosteric interactions in clusters lead to nonlinearity between the input signal and the output kinase activity, allowing amplification and integration of signals and increased dynamic range [14,20–23]. However, while the chemotaxis system controls cell motility, most two-component systems mediate adaptive responses that involve gene expression. Thus, during its evolution, the chemotaxis system was subjected to functional constraints different from those faced by most two-component systems, and receptor clustering in chemotaxis might be a consequence of some of these unique functional constrains. Polar localization of sensory kinases was also demonstrated in the bacteria *Caulobacter crescentus* [24] and *Xanthomonas campestris* [25]. In the former, however, clustering appears to serve a highly specialized function in the asymmetric cell division. In *E. coli*, clustering of the DcuS and CitA sensory kinases [26,27] and the cytoplasmic response regulators OmpR and PhoP [28,29] have been reported. Nevertheless, the physical organization of most sensors remains unclear [30]. Investigating the cellular organization of the canonical sensory kinases is essential not only for understanding the functions of these individual systems but also for getting fundamental insights into the role of clustering in sensory signaling. Here we performed a systematic *in-vivo* study of physical associations and clustering of sensory kinases in *E. coli*, observing prominent tendency for clustering of the TorS and EvgS sensors.

## Results and Discussion

### Preliminary screen for self-association of sensory kinases in *E. coli*

**Figure 1:**
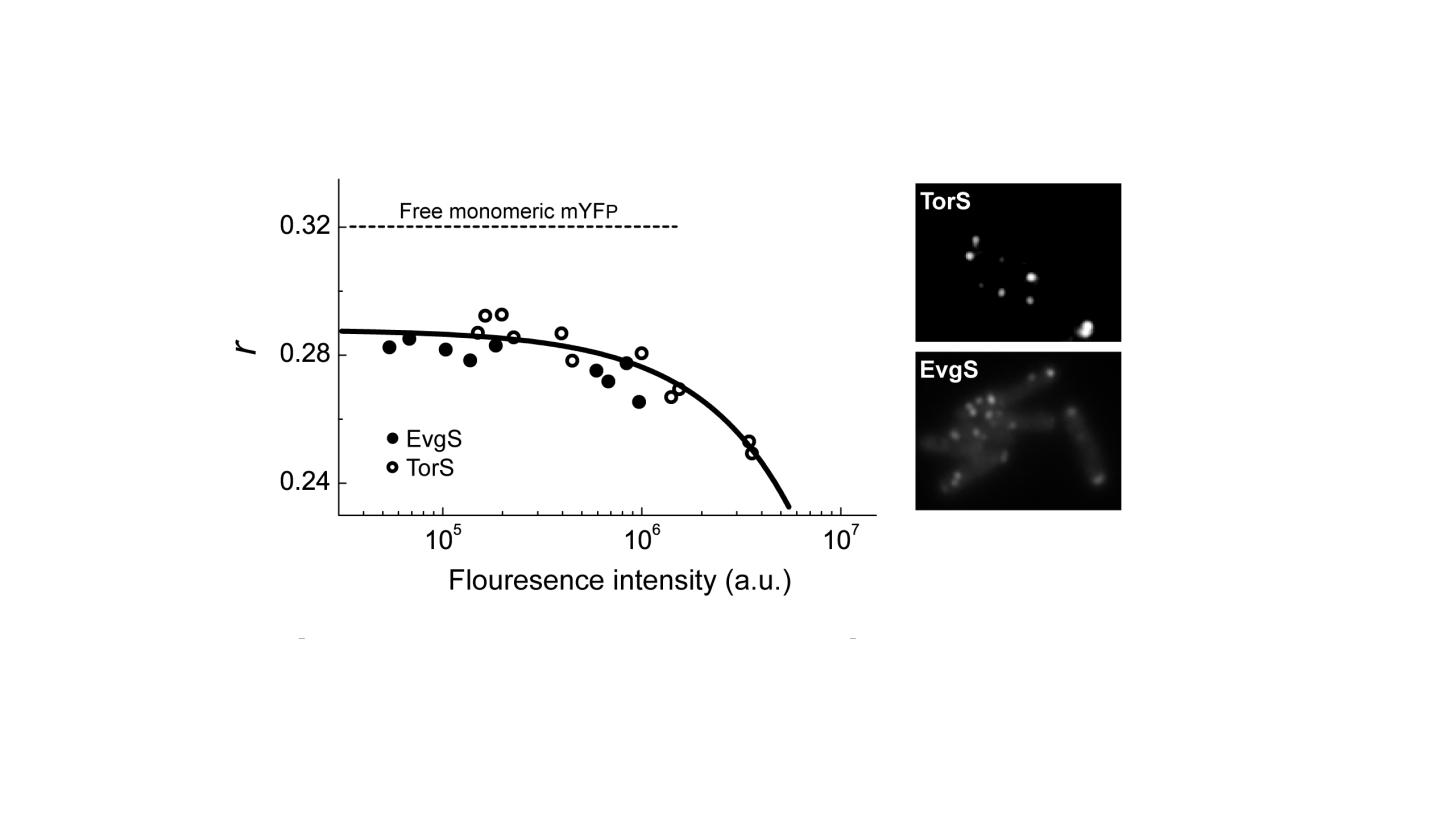
TorS and EvgS show distinct associations and clustering properties. The fluorescence anisotropy (*r*) measured from MG1655 cells expressing mYFP tagged TorS or EvgS sensors at various expression levels, using 0-100 µM IPTG, which was assumed to be correlated with the total fluorescence. A total fluorescence intensity of 10^5^ counts/second corresponds to approximately 4,000 copies of mYFP per cell, estimated as described in Materials and Methods. Line is a guide to the eye. Typical fluorescence images of these cells are shown on the right.

We constructed a plasmid library of C-terminal fusions of monomeric yellow fluorescent protein EYFP^A206K^ (mYFP) to all 27 of the canonical transmembrane sensory kinases in *E. coli*. The cytoplasmic kinases CheA and NtrB and the plasmid-born sensor PcoS were not included in this study. We subsequently excluded the fusions to ArcB and RstB from further analysis since immunobloting analysis using an antibody against mYFP indicated that these fusions were highly degraded. The level of protein degradation of all other receptor fusions used in this study and their expression levels used for the imaging experiments are shown in Table S1. We have used fluorescence images and homo-FRET measurements [22,31] of these tagged sensors expressed in wild-type *E. coli* strain MG1655 to screen for sensors with potentially higher tendency to form clusters (Figure S1, Table S2). Under the growth and imaging conditions used in these experiments, the EvgS and TorS showed the most distinct tendency for self-asociation (low anisotropy) over a wide rang of expression levels and formed high-contrast and robust clusters seen by fluorescence microscopy (Figure 1). Self-association of the TorS and EvgS fusions was further confirmed by spectral-shift FRET, where fusions of the same sensor to monomeric cyan fluorescent protein (mCFP) and mYFP were co-expressed in cells and FRET was detected by acceptor photobleaching (Figure S2, A and B). 

The mYFP is a truly monomeric fluorescence protein with undetectable affinity for self-association [32]. Therefore, we do not expect this tag to directly promote self-association of the tagged sensors. On the other hand, the mYFP tag can potentially inhibit naturally occurring interactions between sensors by steric hindrance. Therefore, by using tagged sensors we are most likely underestimating the tendency of sensors for self-association. Clustering of these sensors can also be underestimated if other components are involved, which would require a proper stoichiometry, or if specific external conditions are required for clustering. In any case, in this report we focused on the clustering properties of the TorS and EvgS sensors, which had the most distinct tendency for clustering under the conditions of our experiments. 

### mYFP-tagged TorS and EvgS cluster at low expression levels.

**Figure 2:**
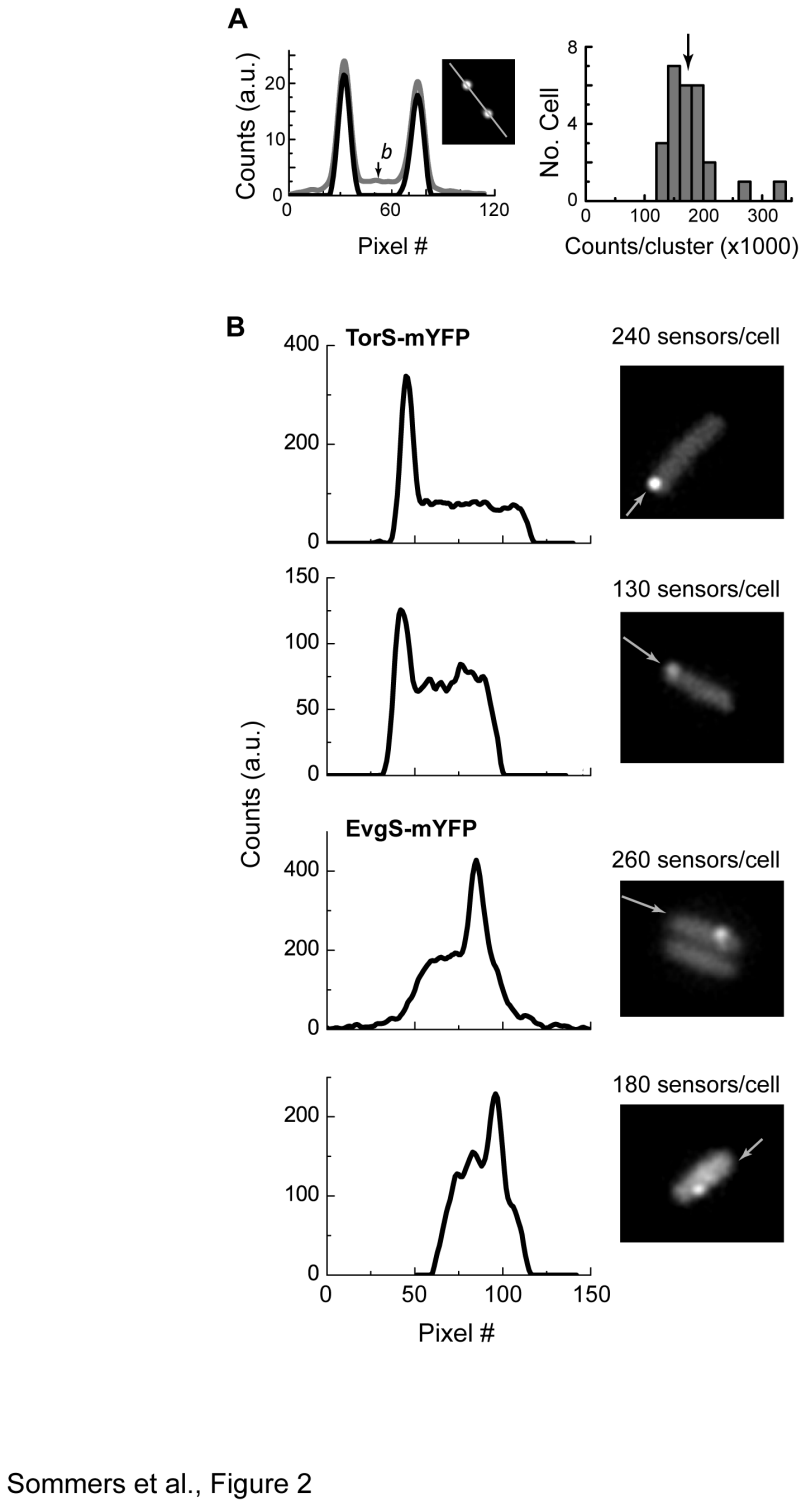
Clustering of mYFP-tagged TorS and EvgS sensors at low expression levels. (A) The general schema used for calibration of the fluorescence intensity (see Materials and Methods). Shown on the left is an image and intensity profile of IL2 cells containing a TetO DNA operator and expressing TetR-mYFP from a pBAD33 vector induced with 0.001% arabinose. Two profiles of the same cell are shown: before (gray line) and after (black line) subtracting the cellular background marked with arrow. Shown on the right is a histogram of the integrated fluorescence intensity per locus obtained from different cells. (B) Fluorescence images of MG1655 cells expressing mYFP-tagged TorS or EvgS and the corresponding intensity profiles taken along the axis marked by the arrow. Fusions were expressed from pBAD33 vector induced with 0.001% arabinose. The sensor expression level (shown above each image) was derived based on the calibration described in (A).

The data presented in Figure 1 indicate that at expression levels of few thousand copies per cell the tagged TorS and EvgS sensors have strong tendency for self-association. This expression level is comparable or below the expression level of the chemoreceptors in *E. coli*, with up to 10,000 copies per cell [33], demonstrating that TorS and EvgS have similar tendency to form clusters as the chemoreceptors. Nevertheless, these expression levels are still above that of the EnvZ sensor, with approximately 100 copies per cell [34]. Thus, we sought to verify the clustering of TorS and EvgS fusions at lower expression levels. By cloning these constructs into a pBAD33 vector under control of the arabinose-inducible pBAD promoter we could considerably lower the expression levels of the sensors; however, the expression levels still varied between cells. Thus, to ensure direct correlation between clustering and expression level, we estimated the amount of sensors expressed in each cell and correlated it directly with clustering in the same cell. The individual expression level of each cell was estimated by comparing the integrated fluorescence from the cell with that obtained from mYFP-tagged TetR repressor localized to a TetO array containing 240 repetitions of the TetR binding motif [35]. The calibration scheme is shown in Figure 2A and explained in detail in Materials and Methods. As shown in Figure 2, mYFP-tagged TorS or EvgS form distinct clusters even in cells that overall contain as few as 150-250 sensors per cell. Thus, despite some uncertainty of the estimate, it indicates that the clustering of these sensors occurs over a wide range of protein levels that is likely to cover most of their potential physiological range.

### Evidence for clustering of the native (untagged) TorS

**Figure 3:**
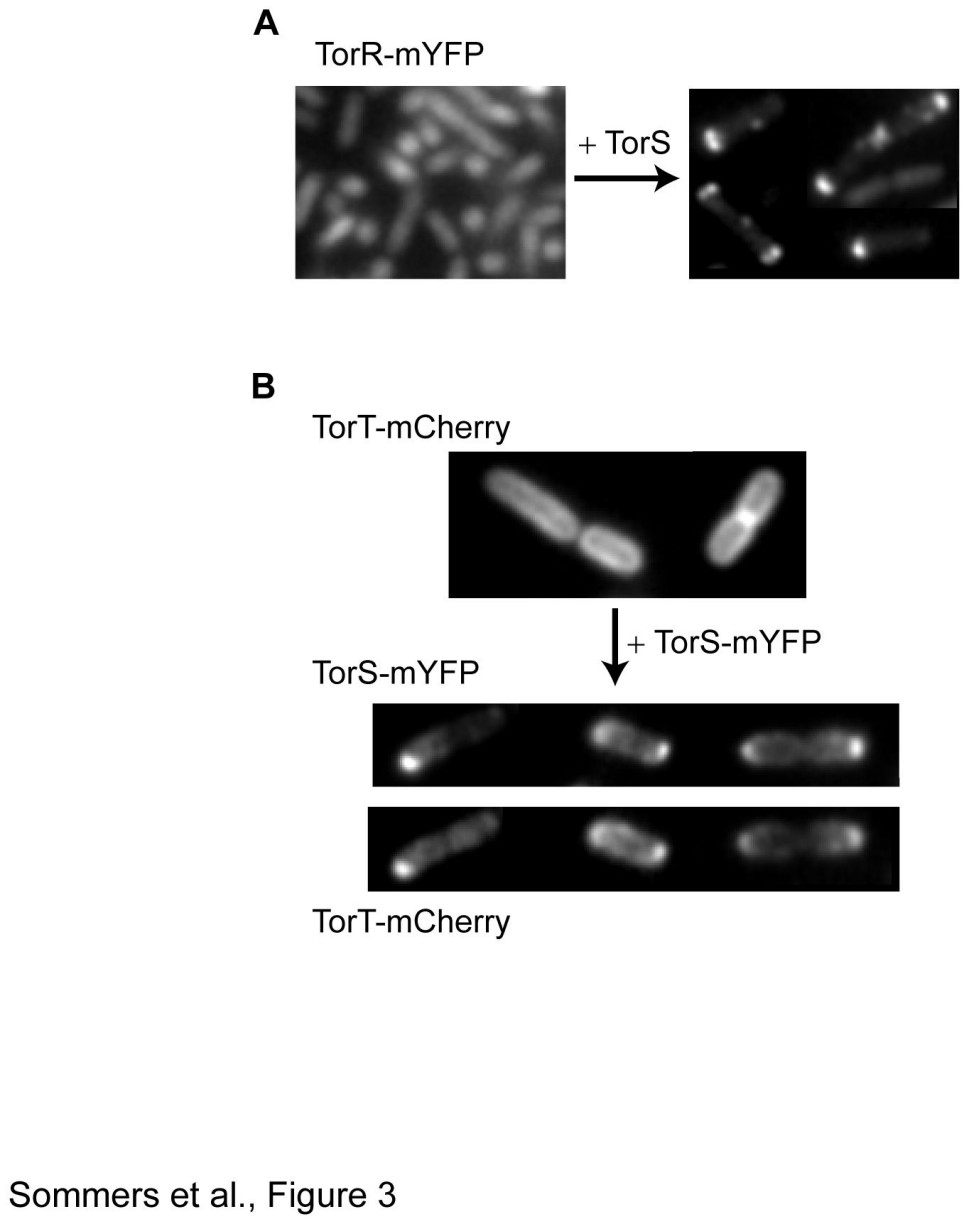
TorS-dependent clustering of TorR and TorT. (A) Fluorescence images of MG1655 cells expressing TorR-mYFP without (left) or with (right) induction of the native (untagged) TorS sensors. (B) Fluorescence images of MG1655 cells expressing TorT-mCherry without (upper part) or with (lower part) induction of TorS-mYFP sensors. The lower part presents images of either the TorS-mYFP or the TorT-mCherry in the same cells.

The association between TorR, the cytoplasmic response regulator, and the membrane-bound TorS sensor can be expected to recruit TorR to TorS clusters. We therefore opted to use this association to verify the clustering of the untagged TorS sensors. We cloned the untagged TorS sensor into the pBAD33 vector and co-expressed it together with TorR-mYFP. When TorS was not induced, the response regulator TorR-mYFP showed homogeneous fluorescence distribution throughout the cell (Figure 3A, left image). This might be due to the insufficient of expression of the native TorS sensors under our experimental conditions and/or due to the reduced sensitivity of this assay compared with direct imaging of the sensors. The latter is expected due to the fact that TorR is a cytoplasmic protein and thus necessarily yields an increased fluorescence background in the cell body from the unbound protein fraction. We therefore increased the levels of the untagged TorS using 0.003% (w/v) arabinose. Induction of the TorS sensor clearly led to clustering of the mYFP-tagged TorR (Figure 3A, right image). Induction of TorS did not lead to clustering of free mYFP or QseC-mYFP, indicating that the TorS-dependent clustering is specific and most likely promoted by the association of the tagged TorR with clusters of the untagged TorS sensors. Such sensor-dependent localization of cytoplasmic components is well known in the case of the chemotaxis system [17] and it was also proposed for the response regulator OmpR [28]. These data confirm that the native untagged TorS sensors also tend to form clusters. Similar experiments done using the untagged EvgS and mYFP-tagged response regulator EvgA were not conclusive because EvgA kept its cytoplasmic homogeneous fluorescence pattern even when sensor was induced. The lack of membrane localization of the tagged EvgA suggests that it is only weakly associated with EvgS. Consistent with that, no interaction between EvgS-mCFP and EvgA-mYFP could be observed by the spectral-shift FRET, whereas a strong interaction was observed between TorS-mYFP and TorR-mCFP (Figure S2, C and D).

### Functionality of the mYFP-tagged TorS and EvgS sensors

**Figure 4:**
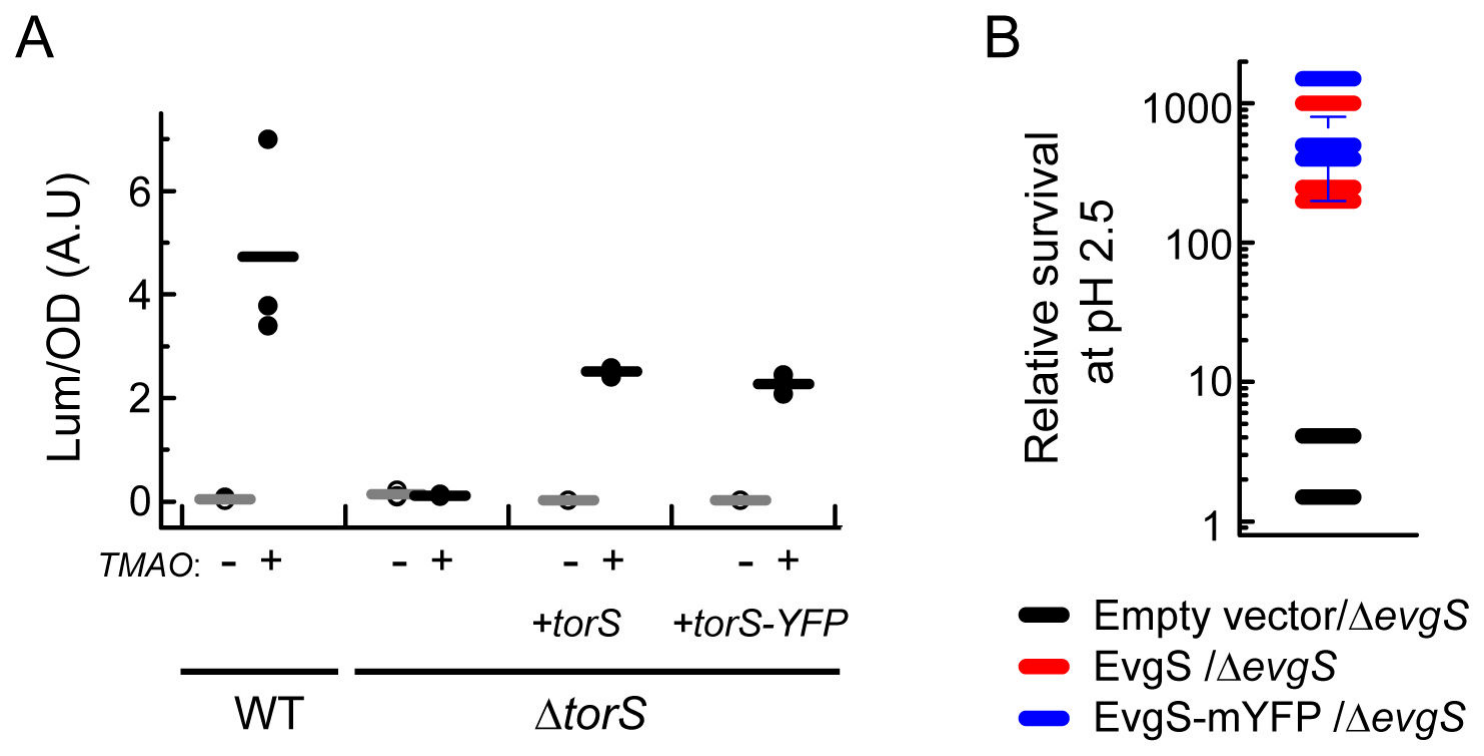
Evidence for functionality of the mYFP-tagged TorS and EvgS sensors. (**A**) Wild-type, Δ*torS*, or Δ*torS* cell transformed with pBAD33 plasmid carrying either *torS* or *torS-mYFP* genes were tested for the TMAO-dependent activity of the *torCAD* promoter, using the *lux* luminescent system (see *Materials* and *Methods*). The raw data for three repetitions of the experiment are shown (symbols), where each point represents the average of two reading in independent wells. Bars represent the average reading of the three experiments. (**B**) Cells were tested for their survival of low-pH stress using a protocol adapted from that used in Ref [38]. Cells were pre-grown at pH 5.5 or 7.6, moved to pH 2.5 for one hour, and then plated on LB plats. An estimated ratio of the surviving cells (pH5.5/pH7.6) is sown for three experiments and three strains: *evgS* deletion strain (black symbols); *evgS* deletion strain complemented with EvgS-mYFP (red symbols); and, *evgS* deletion strain complemented with untagged EvgS (blue symbols). The marked error bar represents the uncertainty in the colony estimate, which was exceptionally large in this experiment.

The fact that untagged TorS tends to form clusters (Figure 3A) suggests that the clustering of tagged TorS is not mediated by the tag. We further checked the integrity and proper folding of the tagged TorS sensor. Association between TorS-mYFP and TorR-mCFP could be observed by the spectral-shift FRET (Figure S2). We then tested whether clustering of TorS-mYFP can drive the localization of the periplasmic binding protein TorT which was shown to bind to TorS [36]. We used wild-type cells expressing TorT-mCherry and TorS-mYFP under inducible promoters. When TorS-mYFP was not induced, TorT-mCherry showed homogeneous peripheral distribution as expected from a periplasmic protein (Figure 3B, upper part). However, upon induction of TorS-mYFP, the TorT-mCherry protein formed clusters that co-localized with those of TorS-mYFP (Figure 3B, lower part). In the absence of mCherry no fluorescence could be detected in the mCherry channel. These data further confirm that TorT is indeed bound to TorS *in vivo*. Taken together, the general integrity and overall proper folding of the tagged TorS sensor is confirmed by its expected associations with the membrane, with the cytoplasmic TorR, and with the periplasmic TorT. We further investigated the ability of the tagged TorS sensors to modulate the activity of *torCAD* operon in response to TMAO (20 mM) by using the *lux* system [37]. *E. coli* strains MG1655 (WT) and JW1535 (Δ*torS*) were transformed with a plasmid carrying the *lux* operon under the control of the *torCAD* promoter. In addition to the *lux* carrying reporter plasmid, the Δ*torS* cells were also transformed with a pBAD33 plasmid carrying *torS* or *torS-mYFP* genes under the control of arabinose promoter. As seen in Figure 4A, while the luciferase activity was clearly higher in the presence of TMAO in the WT strain, no effect of TMAO could be observed in the Δ*torS* deletion strain. However, expressing either the tagged or untagged TorS recovered the TMAO-induced activity of the *torCAD* promoter to a similar level. This evidence further supports the conclusion that the tagged TorS sensor maintains its general functional properties. 

We tested the functionality of tagged EvgS sensors by using the reported role of EvgS in promoting the survival of *E. coli* cells at low pH [38]. This EvgS-mediated survival was shown to be much more effective when cells were pre-grown at a pH of 5.5 but much less in a pH of 7.5. Thus, following a protocol adapted from Ref. [38], we tested the survival of cells challenged by pH 2.5 for one hour. Three *evgS* deletion strains were tested: (i) transduced with an empty vector, (ii) transduced with a vector carrying an untagged EvgS, and (iii) transduced with a vector carrying mYFP-tagged EvgS. We indeed find that the survival rate of cells that were pre-grown at pH 5.5 is more than 100 fold higher than those that were pre-grown at pH 7.5 (Figure 4B). This differential effect was seen when either the untagged EvgS (red symbols) or the mYFP-tagged EvgS (blue symbols) sensors were present, but not with the empty plasmid (grey symbols). Thus, both the tagged and untagged sensors, but not the empty vector, promoted similar differential survival of cells at pH 2.5. These data suggest that the tagged EvgS sensor maintains its function, at least in the context of pH resistance.

### Determinants of TorS and EvgS clustering

**Figure 5:**
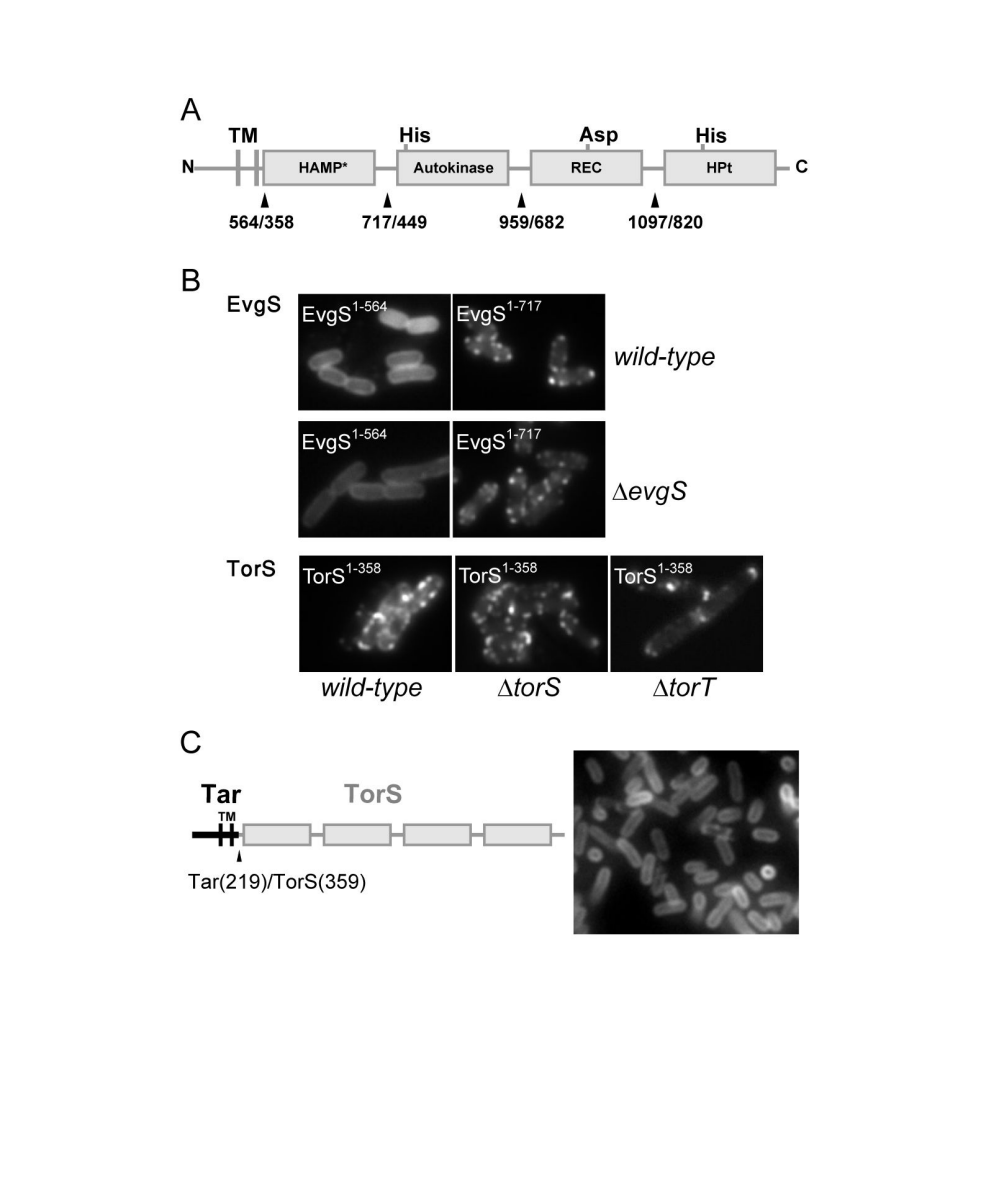
Comparison of the clustering properties of the truncated EvgS and TorS sensors. (**A**) Domain topology of EvgS/TorS hybrid sensor kinases. Marked amino acids indicate positions of YFP fusions in EvgS/TorS, respectively. Abbreviations: TM, transmembrane domains; HAMP, domain found in histidine kinases, adenylyl cyclases, methyl binding proteins, and phosphatases (*verified only in TorS); REC, receiver domain; HPt, histidine containing phosphotransfer domain. (**B**) Fluorescence images of YFP-tagged truncations of EvgS or TorS in wildtype or deletion strains. The truncated fusions of both TorS and EvgS were expressed from the same plasmid under control of a pTrc promoter without inducer, resulting in similar levels of protein expression, which were also similar to those used for the full-length receptors (Figure 1). Images are shown for the shortest segments that showed clustering; longer segments of the sensors showed similar clustering patterns. (**C**) Fluorescence images of mYFP-tagged chimera protein containing the N-terminal transmembrane/periplasmic domain (‘head’) of the Tar chemoreceptor and the C-terminal (cytoplasmic domain) of TorS. The topology of the chimera is shown on the left.

In order to gain insight into the clustering mechanism of the TorS and EvgS proteins, we made a series of C-terminal fusions of mYFP to truncated versions of these sensors, systematically eliminating cytoplasmic portions of these proteins (Figure 5A). We find that by itself the periplasmic domain of EvgS (including the transmembrane part) did not cluster, while the corresponding periplasmic domain of TorS (including the transmembrane part) showed distinct clustering (Figure 5B). Fusions to longer segments of the EvgS sensor showed clusters (Figure 5B). The clustering of the mYFP-tagged TorS periplasmic domain occurred in wild type, ∆*torS*, and ∆*torT* backgrounds (Figure 5B). To further check the role of the cytoplasmic domain of TorS in clustering, we made a chimeric protein containing the periplasmic/membrane domain of the Tar chemoreceptor and the cytoplasmic domain of TorS (Figure 5C, left). These chimeric proteins were recruited to the membrane by the transmembrane part of Tar but mostly showed uniform distribution over the cytoplasmic membrane (Figure 5C, right). It is possible though that changing the periplasmic part of the receptor can in principle affect the conformation of the cytoplasmic domain and thus can affect its clustering properties. Nevertheless, these data is consistent with the observation that the periplasmic domain can promote clustering. Thus, despite the general similarity between the domains structure of EvgS and TorS, with multiple phosphotransfer steps, the clustering determinants of TorS and EvgS appears to be different, whereby the periplasmic/transmembrane part of TorS, but not EvgS, shows tendency for clustering. 

## Conclusions

We studied the self-association and clustering of membrane-bound histidine-kinases sensors in *E. coli*. We show that the TorS and EvgS sensors have a clear tendency for self-association and clustering. Analysing truncated versions of these sensors suggested that clustering of the EvgS sensors relies on interactions between the cytoplasmic domains of the receptors, while clustering of the TorS sensors can be mediated by the periplasmic/transmembrane domains. Overall, these data indicate that TorS and EvgS tend to form clusters in *E. coli* cells, consistent with the notion that higher-order associations between sensory kinases are not limited to the chemotaxis receptors. The functional role of such clustering under native conditions awaits further study.

## Materials and Methods

### Strains and plasmids

Strains and plasmids used in this study are listed in Table S3. All strains are derivatives of the *Escherichia coli* K-12 strain (MG1655). Strains containing deletions of the various HK-sensors were obtained from the Keio knockout collection [39]. Proteins fusions were made using standard molecular cloning techniques. Fluorescent protein fusions were constructed by PCR amplification of the target gene and verified by sequencing and products were cloned into either pDK112 or pDK113 fusion vectors. In both cases, a CCATGGAATTCGAGCTCGGATCCGGAGGTGGA sequence that was placed in front of monomeric *eyfp* ^A206K^ (pDK112) or *ecfp* ^A206K^ (pDK113) allowed cloning of HK-sensors upstream of the tag molecules via *Nco*I and *Bam*HI, resulting in a C-terminal fusion of the tag. CFP fusions were subsequently transferred into pBAD33 via *Spe*I and *Hin*dIII. A majority of the fusions were expressed as full-length proteins with little degradation (Table S1), as veriﬁed using immunoblots with a monoclonal GFP-speciﬁc antibody (JL8; Clontech, Saint-Germain-en-Laye, France). The amount of degradation was determined using public domain software ImageJ 1.40g (Wayne Rasband, http://rsb.info.nih.gov/ij/). Promoter reporters for specific two-component systems were chosen from an *E. coli* promoter library [40] based on promoter description in the EcoCyc database [41].

### Growth conditions and induction levels

Unless specified otherwise, cells were grown in either Tryptone Broth (TB; Bacto Tryptone 1% w/v, NaCl 0.5% w/v), Lysogeny Broth (LB) or minimal A medium (Miller, 1992). Minimal A medium was supplemented with final concentrations of 0.1% w/v casamino acids (Oxide, Basingstoke, UK), 1 mM MgSO_4_ (Merck, Darmstadt, Germany) and 0.2% w/v glucose or glycerol (Applichem, Darmstadt, Germany) as carbon source. For FRET and flow cytometry experiments cells were grown in TB in a rotary shaker at 34°C as described before [17]. Antibiotics ampicillin, kanamycin and chloramphenicol were added to final concentrations of 100 mg/ml, 35 mg/ml and 50 mg/ml, respectively. All overnight cultures were diluted 1:100 and grown until OD_600_ of 0.4–0.5 in the presence of antibiotics and, when appropriate, isopropyl b-D-thiogalactoside (IPTG) or arabinose. Cells from LB or TB cultures were harvested by centrifugation, washed and resuspended in tethering buffer (10 mM potassium phosphate, 0.1 mM EDTA, 1 mM L-methionine, 10 mM sodium lactate, pH 7.0). Cells grown in minimal A medium were washed and resuspended in phosphate buffer (10 mM potassium phosphate, 10 mM sodium lactate, pH 7.0). Before measurements cells were stored at 6°C for about 30 minutes to stop growth and protein expression. 

### Quantification of protein fusions expression level

To estimate mYFP mean expression, reporter protein fluorescence was quantified in ~10.000 cells as described before [42], using flow cytometry on a FACScan (BD Biosciences, Heidelberg, Germany) equipped with a 488-nm argon laser. FACScan data were analysed using CellQuest™ Pro 4.0.1 software (BD Biosciences, Heidelberg, Germany). Expression of mCFP fusions was estimated using fluorescence imaging as described below. Pictures were quantified using public domain software ImageJ. In both cases, autofluorescence background of wild-type cells was subtracted. Fluorescence intensities were recalculated into absolute protein numbers using calibration that was done with purified mYFP and mCFP proteins as described previously [43]. Subsequently, expression levels of all protein fusions were adjusted to a few thousand copies per cell. 

To estimate the single-cell expression levels (Figure 2) we used the strain IL2 containing a TetO DNA operator with 240 repetitions of the TetR binding motif [35]. Expression levels were estimated by comparing the fluorescence from a particular cell with that obtained from a TetO locus bound by TetR-mYFP. IL2 cells containing pBAD33 vector (pBAD promoter inducible by L-arabinose) carrying *tetR-mYFP* fusion were grown under standard conditions supplemented by 0.001% arabinose and imaged in motility buffer-based agarose pad (Figure 2A). In most cases two fluorescence spots were visible on two sides of the cell corresponding to two chromosomal loci containing the TetO operator [35]. Expression of TetR-mYFP in cells lacking the TetO operator showed homogeneous fluorescence and, correspondingly, increasing the expression of the TetR-mYFP in IL2 cells beyond 0.001% arabinose mostly enhanced the background fluorescence in the cells as expected from an excess of TetR-mYFP over the binding sites. The cellular background fluorescence was then subtracted from the image leading to two fluorescence spots confined to proximally 200 pixels each (Figure 2A, black line). The total fluorescence per loci, I_L_, was extracted by integrating the total florescence per cell and dividing by the number of loci. Since the fluorescence associated with the airy-disks around the peaks was practically ignored, the I_L_ value somewhat under estimated the actual emitted fluorescence. Since we are looking for those spots that represent loci that are saturated with TetR-mYFP, we chose to analyze cells that appear to have more intense and well-separated fluorescent spots. A histogram of the I_L_ values obtained from these cells is shown in Figure 2A, demonstrating that most values were centered at I_0_=180,000 counts per locus, which was then used as a standard ruler. In order to make sure that we were not over estimating I_0_ the two clusters with significantly higher I_L_ values were ignored, since these rear loci might represent two, closely placed, loci that contained larger numbers of binding sites. Next, under identical imaging conditions, we imaged cells that do not express fluorescence proteins and estimated the intrinsic background fluorescence from these cells, *I*_*back*_ (approximately 60,000 counts per cell). Finally, we imaged the cells expressing mYFP-tagged TorS or EvgS. For each cell, after subtracting the average background intensity, estimated as the average intensity at a nearby area depleted of cells, we integrated the fluorescence from the cell area to yield *I*_*cell*_. Finally, the number of mYFP copies in each cell was estimated, *N*≈240∙(*I*_*cell*_−*I*_*back*_)/*I*_0_. For the low expression levels, this estimate can be off by up to 50% due to uncertainties in the background subtractions.

For the data described in Figure 1, the expression level of the fusion proteins was estimated as follows. mYFP was purified and its concentration was estimated by the absorbance at 280 nm and Coomassie*-stained gel*. Cells expressing free mYFP were grown to OD_600_ of 0.45 and washed as mentioned above. The average intensity obtained from this cell suspension was compared directly with the fluorescence from the purified mYFP solution. Alternatively, cell lysate was extracted and the fluorescence could again be compared with the purified mYFP solution. These comparisons allowed us to estimate the total concentration of mYFP in this cell-suspension. The corresponding cells density in this suspension was measured by plating them on agar plates after appropriate dilutions, yielding an estimate of the average copy number of mYFP per cell. Cells from this cell suspension were attached to a glass coverslip to form a monolayer (as in a normal cell preparation for the polarization measurements), yielding a correlation between the fluorescence intensity for the cell population and the average mYFP copy number per cell. The average mYFP expression in other cell populations was estimated based on this calibration from the fluorescence intensity obtained from a monolayer of these cells. For each sensor, data was taken a few times on different days, and random errors, including those originate due to fluctuation in the surface coverage, are contributing and represented by the scatter in the date. 

### Fluorescence polarization measurements

Cells were immobilized on a coverslip and mounted in a gold-plated brass flow chamber. The flow chamber was mounted on an aluminum fitting in a Nikon FN 1 microscope equipped with a 40x Plan-Fluor objective (0.75 NA), and 150W xenon lamp (Hamamatsu, Bridgewater, NJ). The mYFP proteins were excited with linearly polarized light using a linear glass polarizer (Edmund Optics, Barrington, NJ), ET508/6x excitation filter (Chroma Technology, Brattleboro, VT), and FF520Di01 dichroic mirror. The fluorescence was collected using a FF01-542/27 emission filter (Semrock, Rochester, NY), and split using a polarizing beam splitter cube (Newport, Irvine, CA). The parallel (*I*_*par*_) and perpendicular (*I*_*per*_) polarizations were monitored with photon counters (H7422P, Hamamatsu, Bridgewater, NJ). The steady-state polarization of the emitted fluorescence is represented here by the fluorescence anisotropy, *r*, defined as (*I*_*par*_ ‑ I_*per*_) / (*I*_*par*_+2*I*_*per*_), where *I*_*per*_ has been corrected for imperfections of the optical system. Validation of the absolute fluorescence anisotropy was done by adjusting the anisotropy recorded from aqueous solution of fluorescein to zero. This calibration yielded an anisotropy level of 0.32 for purified mYFP.

### Fluorescence imaging and FRET

For fluorescence imaging and acceptor photobleaching FRET experiments, cells were grown in TB media as mentioned above and applied to a thin agarose pad (1% agarose in tethering buffer). Imaging was performed on a wide-field Zeiss AxioObserver microscope as described previously [44]. mYFP photobleaching was achieved by a brief 20 s illumination using a 532 nm laser described previously on a custom-modified Zeiss Axiovert 200 microscope [43], and integral CFP fluorescence of a field of several hundred cells was recorded before and after bleaching using photon counters (Hamamatsu) with 1 sec integration time. An increase in the CFP signal of more than 0.5 % due to unquenched donor fluorescence upon photoinactivation of the acceptor indicated a positive FRET pair [43]. 

### Luciferase activity assay

Bacterial strains MG1655 (WT) and JW1535 (Δ*torS*) were transformed with a plasmid carrying the *Lux* protein under the control of the *torCAD* promoter (Amp^R^, provided by Prof. Shimshon Belkin, Hebrew University). For the complementation experiments the *torS* deletion strain was transformed also with pBAD33 plasmid carrying *torS* or *torS -mYFP* under the control of arabinose promoter (Cam^R^). Four types of cells were used in these experiments: WT, Δ*torS*, *torS*/Δ*torS*, and *torS-mYFP*/Δ*torS*. Cells were grown overnight at 37°C in LB, diluted 1:10 into LB and placed in a sealed 5 ml tube. Each cell type was grown with or without 20 mM TMAO. The tubes were then placed at 37°C for 4h after which the optical density (OD) of the culture at 600nm (OD_600_) was measured and 100 µl samples were extracted for measuring the luminescent. Luminescent was measured in 96-well plats using a plate reader (Victor^3^ multilabel counter model 1420). The experiment was repeated three times. 

### Low-pH survival assay

The assay was adapted from the protocol used in Ref. [38]. Three strains were used: *evgS* deletion strain, *evgS* strain complemented with a plasmid carrying *evgS-mYFP* under inducible promoter (pTrc), and *evgS* strain complemented with a plasmid carrying *evgS* under the same promoter. Cells were grown in LB medium (Bacto tryptone 1% w/v, Bacto yeast extract 0.5% w/v , NaCl 1% w/v) at 37°C. Two cultures where grown from each strain at pH 5.5 and 7.5. When the optical density (OD_600_) of the cultures reached 0.75-0.8, a sample of 0.5 ml was extracted from each culture and added to 1.5 ml of pre-warmed LB medium at pH 2.5 and incubated for 1 hour at 37°C. Then, from each culture, samples were plated in a serial dilution on LB plates and incubated for 16 hours. For each strain, the survival of the cells grown at pH 5.5 relative to those grown at pH 7.5 was calculated as the ratio between the numbers of bacterial colonies that grew on the respective LB plats.

## Supporting Information

Figure S1 **Cellular localization of mYFP-tagged sensors.** Fluorescence images of MG1655 cells expressing the different mYFP-tagged sensors. For each sensor, images are shown for cells grown under conditions that yielded the most clear localization pattern (Table S1): LB for BaeS, CitA, CpxA, CusS, EnvZ, HydH, PhoQ, PhoR, RcsC, YfhK, YpdA and YehU, and TB for the remaining sensors. Induction levels were as in Table S1. Exposure times were adjusted to the strength of fluorescence. Sensors were arranged according to their distribution over the cell membrane. (**A**) Homogenous distribution. (**B**) Intermediate punctuated distribution. (**C**) Distinct localization. Scale bar: 2 µM. (**D**) The fluorescence anisotropy (*r*) measured from MG1655 cells expressing mYFP tagged sensors at various expression levels, using 0-100 µM IPTG, which assumed to correlate with the total fluorescence. A total fluorescence intensity of 10^5^ counts/second corresponds to approximately 4,000 copies of mYFP per cell, estimated as described in Materials and Methods. Lines are a guide to the eye.
(PDF)

Figure S2 **FRET measurements by acceptor photobleaching between: (**A**) EvgS-EvgS, (**B**) TorS-TorS, (**C**) EvgS-EvgA (**D**) TorS-TorR.** In these experiments, FRET is being manifested as an increase in cyan-channel emission. Labels represent the change in FRET and the standard errors from three independent experiments. For EvgS-EvgA no change was detected. All fluorescent protein fusions were independently expressed from plasmids under control of pTrc or pBAD promoters.
(PDF)

Table S1 **Sensory kinase fusions used in the experiments shown in Figure S1 are shown.** The degradation level of these fusion proteins (1-not degraded; 0-fully degraded) was estimated using western-blot analysis. The induction levels of the fusions are indicated and the resulting expression levels were estimated by FACS analysis (see Materials and Methods).
(PDF)

Table S2 **Summary of the HK localization for different growth conditions.** YFP-tagged HK sensors were expressed from plasmids in MG1655 cells (wt) or in the corresponding deletion background (ko – Keio collection). An attempt was made to adjust the copy numbers to about 2000 copies per cell. Labels: A – minimal A medium; Gly – glycerol; Glu – glucose. The cellular distribution of the sensors was scored as follows: M – homogenous membrane distribution; M/A – punctuate localization; C – homogenous cytoplasmic distribution; n.d. – not determined; n.a. – not available/no stimulus known.
(PDF)

Table S3 **Strains and plasmids.**
(PDF)
