# Supplementary figures and images for: The Sensory Histidine Kinases TorS and EvgS Tend to Form Clusters in Escherichia coli Cells
Source: PLoS One. 2013 Oct 11;8(10):e77708. doi: 10.1371/journal.pone.0077708 (PMC3795677; doi:10.1371/journal.pone.0077708)

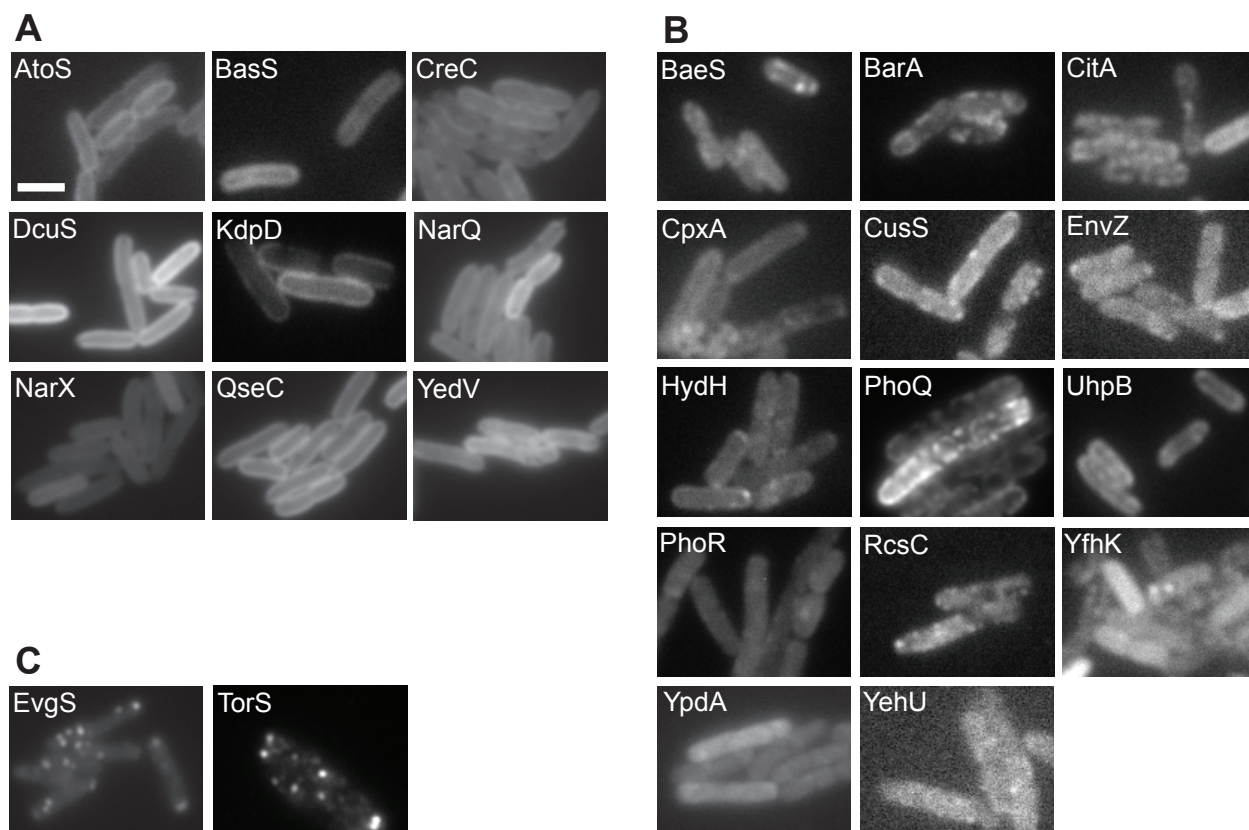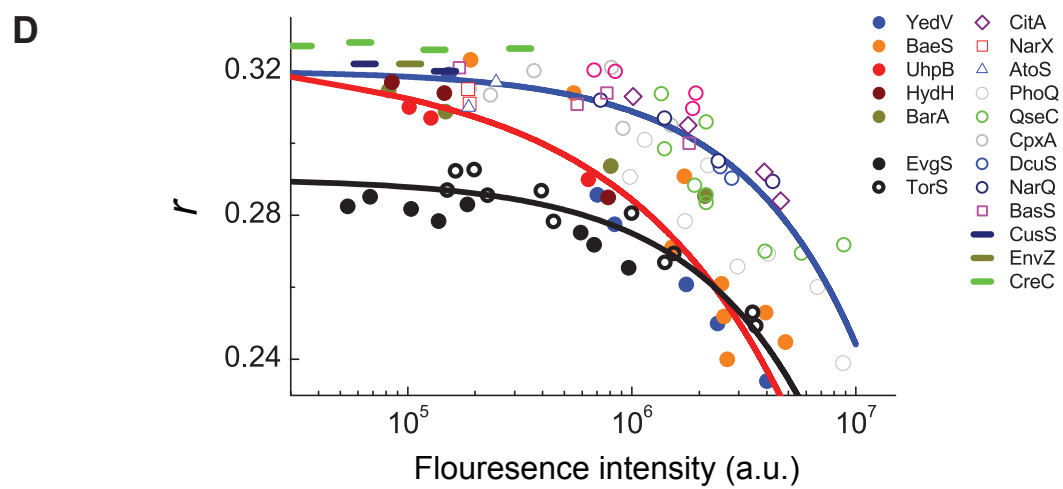

Supplement: Figure S1 — Cellular localization of mYFP-tagged sensors. Fluorescence images of MG1655 cells expressing the different mYFP-tagged sensors. For each sensor, images are shown for cells grown under conditions that yielded the most clear localization pattern (Table S1): LB for BaeS, CitA, CpxA, CusS, EnvZ, HydH, PhoQ, PhoR, RcsC, YfhK, YpdA and YehU, and TB for the remaining sensors. Induction levels were as in Table S1. Exposure times were adjusted to the strength of fluorescence. Sensors were arranged according to their distribution over the cell membrane. (A) Homogenous distribution. (B) Intermediate punctuated distribution. (C) Distinct localization. Scale bar: 2 µM. (D) The fluorescence anisotropy (r) measured from MG1655 cells expressing mYFP tagged sensors at various expression levels, using 0-100 µM IPTG, which assumed to correlate with the total fluorescence. A total fluorescence intensity of 105 counts/second corresponds to approximately 4,000 copies of mYFP per cell, estimated as described in Materials and Methods. Lines are a guide to the eye. (PDF) [file pone.0077708.s001.pdf]

A

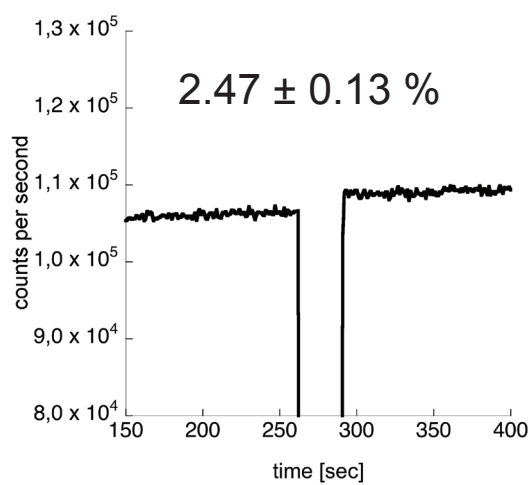

B

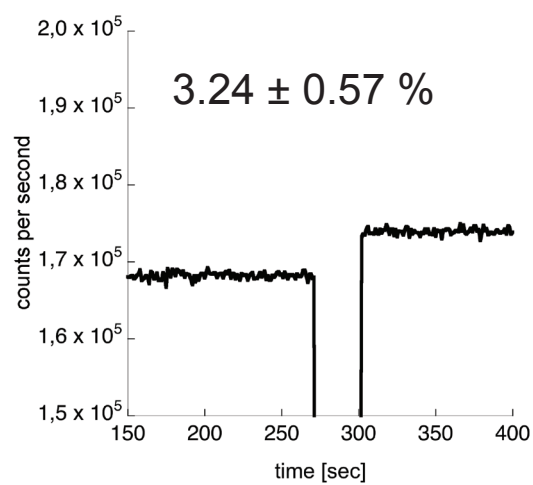

C

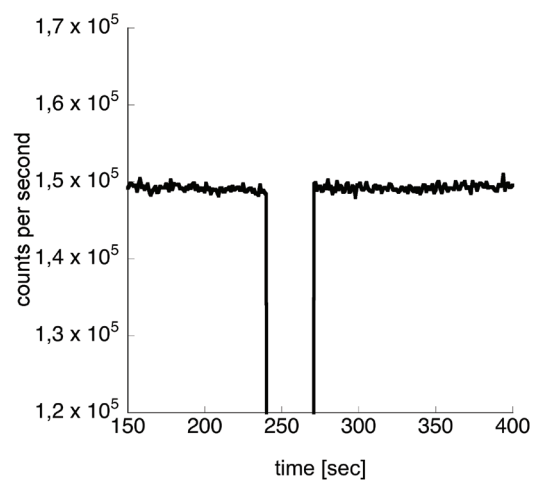

D

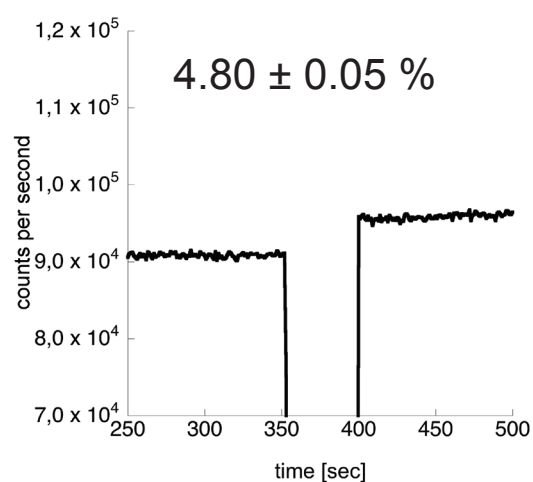

Supplement: Figure S2 — FRET measurements by acceptor photobleaching between: (A) EvgS-EvgS, (B) TorS-TorS, (C) EvgS-EvgA (D) TorS-TorR. In these experiments, FRET is being manifested as an increase in cyan-channel emission. Labels represent the change in FRET and the standard errors from three independent experiments. For EvgS-EvgA no change was detected. All fluorescent protein fusions were independently expressed from plasmids under control of pTrc or pBAD promoters. (PDF) [file pone.0077708.s002.pdf]
