# Supplementary material for: The Sensory Histidine Kinases TorS and EvgS Tend to Form Clusters in Escherichia coli Cells
Source: PLoS One. 2013 Oct 11;8(10):e77708. doi: 10.1371/journal.pone.0077708 (PMC3795677; doi:10.1371/journal.pone.0077708)
Supplement: Table S1 — Sensory kinase fusions used in the experiments shown in Figure S1 are shown. The degradation level of these fusion proteins (1-not degraded; 0-fully degraded) was estimated using western-blot analysis. The induction levels of the fusions are indicated and the resulting expression levels were estimated by FACS analysis (see Materials and Methods). (PDF) [file pone.0077708.s003.pdf]

Sensor	Protein degradetion	Induction level	Copies/cell
AtoS	0.92		
BaeS	0.80	0.005% Ara	8,200
BarA	0.80	0.01% Ara	1,400
BasS	0.90	0 μM IPTG	4,100
CitA	0.98	0 μM IPTG	3,700
CpxA	0.89	0 μM IPTG	2,300
CreC	0.98	7 μM IPTG	2,000
CusS	1.00	30 μM IPTG	2,000
DcuS	0.85	0.005% Ara	7,600
EnvZ	0.7	30 μM IPTG	1,800
EvgS	0.56	0 μM IPTG	7,100
HydH	0.80	3.5 μM IPTG	2,400
KdpD	0.57	0 μM IPTG	24,000
NarQ	0.63	0 μM IPTG	11,300
NarX	0.75	0 μM IPTG	2,700
PhoQ	0.72	0.0001% Ara	21,000
PhoR	0.68	9 μM IPTG	2,000
QseC	0.72	0.0001% Ara	15,800
RcsC	0.66	30 μM IPTG	1,800
TorS	1.00	0 μM IPTG	6,500
UhpB	1.00	0 μM IPTG	1,800
YedV	0.46	0 μM IPTG	12,800
YehU	1.00	30 μM IPTG	2,300
YfhK	1.00	5 μM IPTG	2,000
YpdA	0.55	30 μM IPTG	1,000

Table S1
