# Supplementary material for: The Sensory Histidine Kinases TorS and EvgS Tend to Form Clusters in Escherichia coli Cells
Source: PLoS One. 2013 Oct 11;8(10):e77708. doi: 10.1371/journal.pone.0077708 (PMC3795677; doi:10.1371/journal.pone.0077708)
Supplement: Table S2 — Summary of the HK localization for different growth conditions. YFP-tagged HK sensors were expressed from plasmids in MG1655 cells (wt) or in the corresponding deletion background (ko – Keio collection). An attempt was made to adjust the copy numbers to about 2000 copies per cell. Labels: A – minimal A medium; Gly – glycerol; Glu – glucose. The cellular distribution of the sensors was scored as follows: M – homogenous membrane distribution; M/A – punctuate localization; C – homogenous cytoplasmic distribution; n.d. – not determined; n.a. – not available/no stimulus known. (PDF) [file pone.0077708.s004.pdf]

	LB		TB		Gly/A	Glu/A
YFP fusion	wt	ko	wt	ko	wt	wt
AtoS	M	M	M	n.d.	M	M
BaeS	M/A	M/A	M	M	M	M
BarA	M/A	n.d.	M/A	n.d.	M/A	M/A
BasS	M	M	M	n.d.	M	M
CitA	M/A	M/A	M	M	M	M
CpxA	M/A	M/A	M	M	M	M
CreC	M	M	M	M	M	M
CusS	M/A	M/A	n.d.	n.d.	M	M
DcuS	M	M	M	M	M	M
EnvZ	M/A	M/A	M	M	M	M
EvgS	M/A	n.d.	M/A	M/A	M/A	M/A
HydH	M/A	M/A	M	M	M/A	M
KdpD	M	n.d.	M	n.d.	M	M
NarQ	M	M	M	M	M	M
NarX	M	M	M	M	M	M
PhoQ	M/A	M/A	M	n.d.	M	M
PhoR	M/A	M/A	C	C	M/A	C
QseC	M	M	M	M	M	M
RcsC	M/A	C	M	M	M/A	M/A
TorS	M/A	n.d.	M/A	M/A	M/A	M/A
UhpB	M/A	M/A	M/A	M/A	M	M
YehU	M,M/A	n.d.	C	n.d.	M/A	C
YedV	M	M	M	n.d.	M	M
YfhK	M/A	M/A	M	n.d.	M/A	M
YpdA	M/A	M/A	M	M	M/A	M

Table S2
