# Supplementary material for: The Sensory Histidine Kinases TorS and EvgS Tend to Form Clusters in Escherichia coli Cells
Source: PLoS One. 2013 Oct 11;8(10):e77708. doi: 10.1371/journal.pone.0077708 (PMC3795677; doi:10.1371/journal.pone.0077708)
Supplement: Table S3 — Strains and plasmids. (PDF) [file pone.0077708.s005.pdf]

Table S3

Strain or plasmid	Relevant genotype	Source or reference
Strains		
MG1655	Wild type *Escherichia coli*	Blattner *et al.* , 1996
JW0390	Δ*phoR::kan*^{R}	Baba *et al.* , 2006
JW0611	Δ*citA::kan*^{R}	Baba *et al.* , 2006
JW0979	Δ*torT::kan*^{R}	Baba *et al.* , 2006
JW0980	Δ*torR::kan*^{R}	Baba *et al.* , 2006
JW1115	Δ*phoQ::kan*^{R}	Baba *et al.* , 2006
JW1213	Δ*narX::kan*^{R}	Baba *et al.* , 2006
JW1601	Δ*rstB::kan*^{R}	Baba *et al.* , 2006
JW1951	Δ*yedV::kan*^{R}	Baba *et al.* , 2006
JW2063	Δ*baeS::kan*^{R}	Baba *et al.* , 2006
JW2213	Δ*atoS::kan*^{R}	Baba *et al.* , 2006
JW2366	Δ*evgA::kan*^{R}	Baba *et al.* , 2006
JW2367	Δ*evgS::kan*^{R}	Baba *et al.* , 2006
JW2453	Δ*narQ::kan*^{R}	Baba *et al.* , 2006
JW2994	Δ*qseC::kan*^{R}	Baba *et al.* , 2006
JW3367	Δ*envZ::kan*^{R}	Baba *et al.* , 2006
JW3643	Δ*uhpB::kan*^{R}	Baba *et al.* , 2006
JW3840	Δ*ntrB (glnL)::kan*^{R}	Baba *et al.* , 2006
JW3882	Δ*cpxA::kan*^{R}	Baba *et al.* , 2006
JW3967	Δ*hydH (zraS)::kan*^{R}	Baba *et al.* , 2006
JW4073	Δ*basS::kan*^{R}	Baba *et al.* , 2006
JW4086	Δ*dcuS::kan*^{R}	Baba *et al.* , 2006
JW4362	Δ*creC::kan*^{R}	Baba *et al.* , 2006
JW5082	Δ*cusS::kan*^{R}	Baba *et al.* , 2006
JW5135	Δ*torS::kan*^{R}	Baba *et al.* , 2006
JW5388	Δ*ypdA::kan*^{R}	Baba *et al.* , 2006
JW5407	Δ*yfhK::kan*^{R}	Baba *et al.* , 2006
JW5536	Δ*arcB::kan*^{R}	Baba *et al.* , 2006
JW5917	Δ*rcsC::kan*^{R}	Baba *et al.* , 2006
Plasmids		
pAE-1	CusS-YFP expression plasmid; pDK112 derivative	This work
pBAD33	Expression vector; pACYC ori, pBAD promotor, cam^R	Guzman *et al.* , 1995
pDK112	Expression vector for cloning of C-terminal YFP fusions /pTrc99a	D. Kentner, personal gift
pDK113	Expression vector for cloning of C-terminal CFP fusions /pTrc99a	D. Kentner, personal gift
pES20	BaeS-YFP expression plasmid; pDK112 derivative	This work
pES21	UhpB-YFP expression plasmid; pDK112 derivative	This work
pES22	PhoR-YFP expression plasmid; pDK112 derivative	This work
pES23	RcsC-YFP expression plasmid; pDK112 derivative	This work
pES24	QseC-YFP expression plasmid; pDK112 derivative	This work
pES25	DcuS-YFP expression plasmid; pDK112 derivative	This work
pES26	RstB-YFP expression plasmid; pDK112 derivative	This work
pES27	EnvZ-YFP expression plasmid; pDK112 derivative	This work
pES28	CpxA-YFP expression plasmid; pDK112 derivative	This work
pES29	YedV-YFP expression plasmid; pDK112 derivative	This work
pES30	HydH-YFP expression plasmid; pDK112 derivative	This work
pES31	YpdA-YFP expression plasmid; pDK112 derivative	This work
pES32	NarQ-YFP expression plasmid; pDK112 derivative	This work
pES33	NarX-YFP expression plasmid; pDK112 derivative	This work
pES34	CreC-YFP expression plasmid; pDK112 derivative	This work
pES35	NtrB-YFP expression plasmid; pDK112 derivative	This work
pES37	CitA-YFP expression plasmid; pDK112 derivative	This work
pES38	BasS-YFP expression plasmid; pDK112 derivative	This work
pES39	YfhK-YFP expression plasmid; pDK112 derivative	This work
pES40	AtoS-YFP expression plasmid; pDK112 derivative	This work
pES41	KdpD-YFP expression plasmid; pDK112 derivative	This work
pES42	TorS-YFP expression plasmid; pDK112 derivative	This work
pES43	PhoQ-YFP expression plasmid; pDK112 derivative	This work

pES44	ArcB-YFP expression plasmid; pDK112 derivative	This work
pES45	LytS-YFP expression plasmid; pDK112 derivative	This work
pES46	BarA-YFP expression plasmid; pDK112 derivative	This work
pES47	BaeS-CFP expression plasmid; pDK113 derivative	This work
pES50	EvgS-YFP expression plasmid; pDK112 derivative	This work
pES51	QseC-CFP expression plasmid; pDK113 derivative	This work
pES52	DcuS-CFP expression plasmid; pDK113 derivative	This work
pES70	TorS-CFP expression plasmid; pDK113 derivative	This work
pES71	PhoQ-CFP expression plasmid; pDK113 derivative	This work
pES78	EvgS-CFP expression plasmid; pDK113 derivative	This work
pES102	TorS-CFP expression plasmid; pBAD33 derivative	This work
pES172	EvgS-CFP expression plasmid; pBAD33 derivative	This work
pES113	EvgA-YFP expression plasmid; pDK112 derivative	This work
pES107	TorR-YFP expression plasmid; pDK112 derivative	This work
pES177	$TorS^{1-358}$-YFP expression plasmid; pDK112 derivative	This work
pES181	$EvgS^{1-564}$-YFP expression plasmid; pDK112 derivative	This work
pES182	$EvgS^{1-717}$-YFP expression plasmid; pDK112 derivative	This work
pAV198	Tar1-219-Tor359-end-YFP expression plasmid /pTrc99a	This work
pAVM176	TorS-YFP expression plasmid; pBAD33 derivative	
pAVM159	TorS expression plasmid; pBAD33 derivative	
lux-a.pk054.f7	P_*torCAD-lux* , pBR2TTS derivative, ampR	S. Belkin's Lab
pTrc99a	Expression vector, pBR ori, pTrc promotor, amp^R	Amann *et al.* , 1988
